# Exercise Endocrinology: “What Comes Next?”

**DOI:** 10.3390/endocrines2030017

**Published:** 2021-06-29

**Authors:** Anthony C. Hackney, Kirsty J. Elliott-Sale

**Affiliations:** 1 Department of Exercise & Sport Science, Department of Nutrition, University of North Carolina, Chapel Hill, NC 27599-8700, USA; 2 Musculoskeletal Physiology Research Group, Sport Health and Performance Enhancement (SHAPE) Research Centre, Nottingham Trent University, Nottingham NG11 8NS, UK;

## 1. Introduction

Endocrinology is a branch of physiology medical science that many exercise scientists are embracing in their research pursuits, so much so that “exercise endocrinology” is now viewed by many as a viable subdiscipline in the field. The exponential growth of interest in the interplay between exercise and the hormonal constituents of the endocrine system has resulted in this Special Issue of the journal *Endocrines*.

Historically, peer-reviewed articles addressing elements of exercise and hormones can be traced back to the 1940s, but in these early years, only one to two articles appeared annually, principally due to technological challenges of measuring hormones in those times [1]. In comparison, in 2020 alone, PubMed reported that more than 1000 such articles were published. While the phrase “exercise endocrinology” is still relatively new, the systematic and advanced study of the topic began in earnest in the late 1960s, shortly after the advancement of the radioimmunoassay analytical technique, allowing for a more accurate assessment of hormonal substances in humans [1]. One of the pioneering researchers in this field was Dr. Atko Viru of Tartu University in Estonia (throughout most of his career, Estonia was part of the Soviet Union). Dr. Viru’s research involved over 500 publications leading to groundbreaking discoveries about the responses of hormones to acute and chronic exercise exposure, as well as the mechanisms explaining why such responses occurred in the physiologic “internal milieu” [2]. Subsequently, another leading figure conducting landmark research during the 1970s and 1980s was Dr. John Sutton of Australia. He dynamically pursued many facets of the role of exercise in causing perturbations in circulating hormones. These researchers were quickly followed by Drs. Ann Loucks and Michelle Warren, whose instrumental research on female athletes ultimately lead to the etiology of the Female Athlete Triad diagnosis being elucidated by the medical community in the 1990s [3]. Dozens more innovative researchers, such as Drs. Henrik Galbo, Per Kristian Opstad, Alex Urhausen, Naama Constantini, William Kraemer, Katarina Borer, and Stuart Phillips, to name a few, should be mentioned and discussed for their significant contributions, but space does not allow extensive discussion. Through their diligent work and insights, these investigators provided guidance and examples of the proper scientific approach to conducting research in exercise endocrinology.

This rich research history over the last 60 years by leading scientists on the roles of hormones, exercise, and training is inspirational. However, reflecting on the past naturally leads one to think about the future, which begs the question—”What comes next?” That is, what lies ahead in the future study of exercise endocrinology and needs to be addressed by the next generation of scientists? This editorial paper allows us to provide our perspective on this question, suggest where gaps exist in the research, and make some recommendations for means to advance the study of hormones, exercise, and the endocrine system.

## 2. Proposition One

In conducting exercise studies, there are three basic questions that could be addressed related to hormonal measurements and responses:

- *What happens?* How do hormones change in response to an exercise session?
- *Why does it happen?* What is the physiological role of the hormonal response in the exercise scenario, and is there a measurable consequence (i.e., downstream events)?
- *How does it happen?* Mechanistically, what are the means for regulating and inducing the responses?

Many contemporary exercise studies have focused on the “what happens” question. This is a worthwhile question, but it is also a question already extensively addressed and well-defined for many hormones in a multitude of exercise situations. That is to say, some researchers are addressing an already well-answered question.

Continually answering the same question over and over results in duplication and redundancy of information in the field. It is also “safe science”, as it allows researchers to have high levels of external validity of their findings. There is unquestionably a need for the replication of studies in science to substantiate the robustness of previous findings. However, when it is carried to excess, it undermines one of the key principles of the Scientific Method—acquiring new knowledge.

Consequently, there is a need for researchers to readjust their focus and research aims toward aspects of questions two and three noted above—in other words, to provide some context as to why and how changes in hormones occur with exercise [4]. These questions, however, are more difficult to address in research designs and require more advanced technical procedures and approaches within methodologies. Nevertheless, studies incorporating and approaching aspects of these questions will provide much-needed insight into the understanding of the means by which the endocrine system enables and regulates the adaptation process, especially with respect to exercise training. This seems especially true with responses related to molecular endocrinology and the epigenetic influences of exercise on the endocrine system. New research along these lines will provide greater insight into how exercise can be used more effectively as a means to modify disease states and their associated comorbidities, i.e., providing more understanding as to how “exercise is medicine”.

## 3. Proposition Two

Clinical reference values for hormones exist for a myriad of situations in humans, i.e., children, adolescents, the elderly, males, females, pathological, non-pathological, etc., but they do not exist for athletic, highly trained individuals. Exercise training exhibits powerful and dynamic influences on the human body at rest and in response to physical activity, and the endocrine system is not exempt from these effects. Yet, when clinical evaluations are conducted in athletic populations, standard clinical norms are nearly universally used to assess whether the hormonal values are normal or abnormal. This practice presents the opportunity for the medical misdiagnosis of athletes and potentially incorrect treatments. Steps need to be taken to examine the ability of exercise training to impact circulating levels of hormones in athletes, profile healthy reference ranges, and determine what acceptable nadir and apogee values to expect for such men and women. The recent review by Handelsman et al. concerning testosterone laid such groundwork for this anabolic hormone in athletes [5], but an expanded repertoire of hormones needs to be evaluated, and determinations must be made in a similar fashion.

## 4. Proposition Three

In the simplest terms, the sexes can be divided into males and females, although in our opinion, females need to be further categorized based on their principal sex hormone profiles, namely estrogen and progesterone. The three most general classifications are eumenorrheic females, females with menstrual irregularities, and hormonal contraceptive users, with each group having distinct hormonal milieus. “Hormonal contraceptives” is an umbrella term used to describe implants, injections, pills, patches, vaginal rings, and intrauterine systems, which significantly alter the endogenous concentrations of estrogen and progesterone. “Menstrual irregularities” is also a hypernym, which includes conditions such as amenorrhea, oligomenorrhea, anovulation, and luteal phase deficiency, again rep-resenting distinctive ovarian hormonal profiles. The future of exercise endocrinology relies on researchers investigating males and females to a similar extent (contrary to the historical sex bias in favor of males) and including females from each of the three classifications to increase the generalization of their findings. In elite female athletes, there is an almost 50/50 split between hormonal contraceptive users and non-users (i.e., eumenorrheic females plus those with menstrual irregularities) [6]. It is widely accepted that the prevalence of menstrual disorders/irregularities is higher in the athletic population in comparison with the general population [7]. The lack of inclusion of females as participants in sports and exercise studies clearly demonstrates a currently under-investigated and under-performing area of exercise endocrinology.

## 5. Proposition Four

The field of endocrinology is constantly expanding the number and type of hormones found in humans. Depending upon which reference source one examines, there are currently over 60 known hormones associated with human endocrine glands. This list is evolving and expanded upon regularly. Researchers in exercise endocrinology need to be cognizant of these new chemical messengers and their potential roles in affecting exercise capacity and adaptations in response to training. That said, the effect of exercise on some of the most recently discovered hormones is either not understood at all or no consensus has been developed since so few studies exist. Hence, for these hormones, the issues noted in Proposition One are critical. To overlook or ignore these new hormones, and/or not to anticipate that additional new hormones will be identified in the future, will limit or be a major omission that impedes exercise endocrinology research [8].

## 6. Conclusions

Our intent in this editorial is to provide guidance and direction on how the advancing study of hormones and exercise can lead to better scientific evidence on how to improve the performance, health, and well-being of athletes and exercisers. Obviously, the recommendations herein are not an exhaustive list, but we view them as being of major importance and hopefully thought-provoking. We encourage researchers pursuing exercise endocrinology to carefully consider them. Looking ahead, it is an exciting time for researchers in exercise endocrinology as the area evolves and expands upon the work of the last 60 years. We are certain the future of this dynamic and evolving field will be rich in new insights on human adaptation to exercise, ultimately leading to better health and greater performance.

## References

[1] Lepage R; Albert C Fifty years of development in the endocrinology laboratory. Clin. Biochem 2006, 39, 542–557. 16730257 10.1016/j.clinbiochem.2006.03.007

[2] Hackney AC; Lane AR Exercise endocrinology: Guidance for future research direction and focus. J. Steroids Horm. Sci 2015, 6, e114. 29657899 10.4172/2157-7536.1000.e114 PMC5896280

[3] Constantini NW; Warren MP Special problems of the female athlete. Baillière’s Clin. Rheumatol 1994, 8, 199–219. 8149444 10.1016/s0950-3579(05)80232-8

[4] Johnson EO; Kamilaris TC; Chrousos GP; Gold PW Mechanisms of stress: A dynamic overview of hormonal and behavioral homeostasis. Neurosci. Biobehav. Rev 1992, 16, 115–130. 1630726 10.1016/s0149-7634(05)80175-7

[5] Handelsman DJ; Hirschberg AL; Bermon S Circulating testosterone as the hormonal basis of sex differences in athletic performance. Endocr. Rev 2018, 39, 803–829. 30010735 10.1210/er.2018-00020 PMC6391653

[6] Martin D; Sale C; Cooper SB; Elliott-Sale KJ Period prevalence and perceived side effects of hormonal contraceptive use and the menstrual cycle in elite athletes. Int. J. Sports Physiol. Perform 2018, 13, 926–932. 29283683 10.1123/ijspp.2017-0330

[7] American Academy of Pediatrics. Committee on sports medicine and fitness. Medical concerns in the female athlete. Pediatrics 2000, 106, 610–613. 10969112

[8] Higham JP Field endocrinology of nonhuman primates: Past, present, and future. Horm. Behav 2016, 84, 145–155. 27469069 10.1016/j.yhbeh.2016.07.001

